# Facet joint arthritis as the presenting symptom for culture-negative *Aggregatibacter aphrophilus* native valve endocarditis in a patient without known cardiac disease: a case report

**DOI:** 10.1186/s12879-025-10913-7

**Published:** 2025-05-08

**Authors:** Yurika Okuyama, Koki Kikuchi, Samuel David Stephenson, Naritomo Nishioka, Takahiro Doi, Junya Yamagishi, Satoshi Yuda

**Affiliations:** 1 Department of General Internal Medicine, Teine Keijinkai Hospital, Sapporo, Japan; 2 Department of Infectious Diseases, Teine Keijinkai Hospital, Sapporo, Japan; 3 Department of Internal Medicine, University of Texas Medical Branch, Galveston, USA; 4 Department of Cardiovascular Surgery, Teine Keijinkai Hospital, Sapporo, Japan; 5 Department of Cardiology, Teine Keijinkai Hospital, Sapporo, Japan; 6 International Institute for Zoonosis Control, Hokkaido University, Sapporo, Japan; 7 One Health Research Center, Hokkaido University, Sapporo, Japan

**Keywords:** *Aggregatibacter aphrophilus*, Culture-negative infective endocarditis., 16S rRNA broad-range PCR, Facet joint arthritis

## Abstract

**Background:**

*Aggregatibacter aphrophilus* (*A. aphrophilus*) is a rare cause of infective endocarditis (IE), but is a recognized cause of culture-negative IE. The risk of developing IE is increased in patients with valvular disease or prosthetic valves. To our knowledge, *A. aphrophilus* has never previously been reported to cause lumbar facet joint arthritis in combination with IE.

**Case presentation:**

We present the first case where facet joint arthritis was the presenting symptom for culture-negative *A. aphrophilus* native valve IE in a patient with no prior cardiac disease. A 58-year-old Japanese male without known cardiac disease, presented with high fever, chills, and lower back pain. Initial laboratory evaluation showed leukocytosis and transaminitis. Transthoracic echocardiography revealed an aortic valve vegetation with moderate aortic regurgitation. Magnetic resonance imaging (MRI) showed high-intensity areas in the right iliopsoas muscle and L4/L5 facet joint, indicative of fluid accumulation and disseminated lesions. Multiple sets of blood cultures showed no bacterial growth. Broad-range polymerase chain reaction (br-PCR) for 16S ribosomal RNA on both blood and hepatocytes (due to the patient’s acute liver damage) also failed to identify the causative organism. The patient developed heart failure, and transesophageal echocardiography showed severe aortic regurgitation and an aneurysm at the noncoronary cusp of the aortic valve with perforation. He underwent aortic valve replacement and his symptoms were promptly improved. Although cultures from the excised valve were negative, br-PCR on the valve tissue eventually confirmed the presence of *A. aphrophilus*.

**Conclusion:**

This is the first reported case of culture-negative native valve IE caused by *A. aphrophilus* presenting with facet joint arthritis in a patient without known cardiac disease. Our case emphasizes the importance of considering IE in patients with fever and unexplained musculoskeletal pain, even without known cardiac disease. When conventional diagnostic tests are inconclusive, br-PCR on excised valve tissue is indispensable. Further improvements in non-invasive diagnostic methods are needed to facilitate early diagnosis and treatment.

## Background

*Aggregatibacter aphrophilus* (*A. aphrophilus*), a member of the HACEK group (*Haemophilus* species, *Aggregatibacter* species, *Cardiobacterium hominis*, *Eikenella corrodens*, and *Kingella* species), is a rare cause of infective endocarditis (IE) but is notably implicated in culture-negative forms of the disease [1]. In culture-negative IE, pathogens are often undetectable by conventional culturing methods but can be identified using broad-range polymerase chain reaction (br-PCR), a technique that enhances detection by amplifying bacterial DNA directly from clinical samples [2]. This specific phenomenon has occasionally been reported for *A. aphrophilus* IE, with br-PCR of the excised valve being the only positive diagnostic tool when every other diagnostic method, including cultures of the excised valve, remained negative [3, 4]. Culture-negative *A. aphrophilus* IE has also been reported to have disseminated lesions, specifically shoulder and brain abscesses [5, 6]. *A. aphrophilu*s IE typically affects damaged or bioprosthetic valves; however, there are rare cases of *A. aphrophilus* IE in patients with healthy native heart valves [7]. Lastly, *A. aphrophilus* has been reported to cause vertebral arthritis and abscesses in the absence of IE [8]. To the best of our knowledge, *A. aphrophilus* has never previously been reported to cause lumbar facet joint arthritis in combination with IE. We present a novel case where facet joint arthritis was the presenting symptom for culture-negative *A. aphrophilus* IE.

### Case Presentation

A 58-year-old Japanese male presented with a five-day history of high fever, chills, and back pain. Initial laboratory evaluation by the patient’s primary care physician showed leukocytosis and transaminitis, prompting admission to our hospital for suspected acute liver injury. His medical history included hypertension, dyslipidemia, and a previous stroke, but no known cardiovascular diseases. Additionally, he had no recent history of dental procedures, immunosuppressant use, antibiotic therapy, or significant contact with animals or insects.

His physical examination revealed jaundice and dental issues. Auscultation revealed a II/VI diastolic murmur loudest in the left third intercostal space. The right lumbar region was tender to palpation and the Patrick test was positive, suggesting musculoskeletal involvement. There were no signs of retinal hemorrhages, Osler nodes, or Janeway lesions. The patient had a persistent fever, up to 38.8℃ with other vitals remaining stable.

Initial laboratory evaluation revealed leukocytosis to 13,530/μL, thrombocytopenia to 18,000/μL, prolonged prothrombin time (PT) of 14.8 s, transaminitis with aspartate aminotransferase (AST) at 294 U/L, and alanine aminotransferase (ALT) at 137 U/L. Renal function was near normal with blood urea nitrogen (BUN) at 28.6 mg/dL and creatinine (Cre) at 0.85 mg/dL. C-reactive protein (CRP) was elevated at 9.28 mg/dL, and rheumatoid factor (RF) was positive at 30 IU/mL. Urinalysis showed no hematuria or proteinuria.

*(Note: Although the RF was positive, the patient had no history of chronic joint pain or recurrent fever; this finding was interpreted as a nonspecific inflammatory response associated with IE.)*

**Fig. 1:**
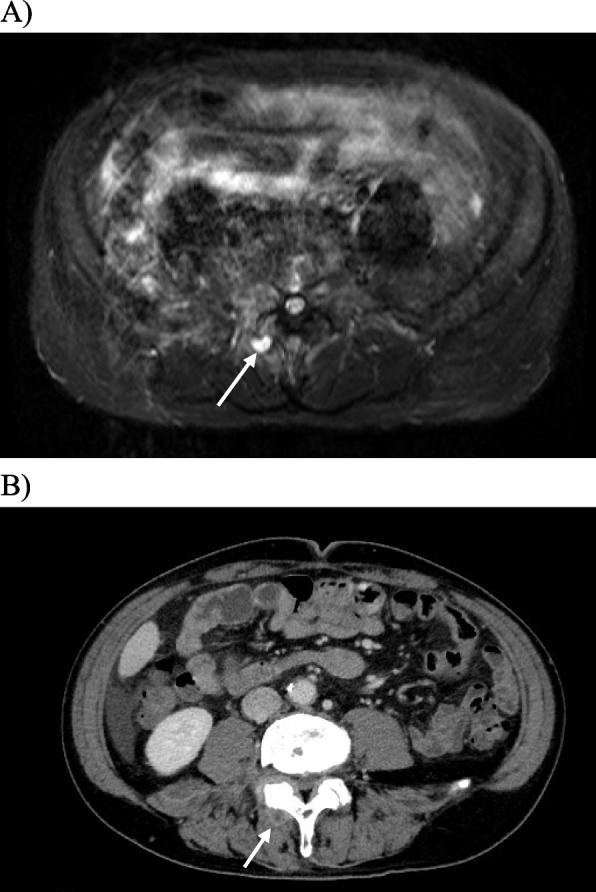
Image findings. **A** MRI showed high signal intensity areas in the right iliopsoas muscle and L4/L5 facet joint under STIR conditions, suggestive of fluid accumulation, indicative of inflammation, possibly pointing to disseminated lesions. **B** Computed tomography (CT) image at the same level

Magnetic resonance imaging (MRI) conducted to investigate his persistent lower back pain revealed high-signal intensity areas in the right iliopsoas muscle and L4/L5 facet joint under short tau inversion recovery (STIR) conditions, indicating inflammation and potential fluid accumulation (Fig. 1).

**Fig. 2:**
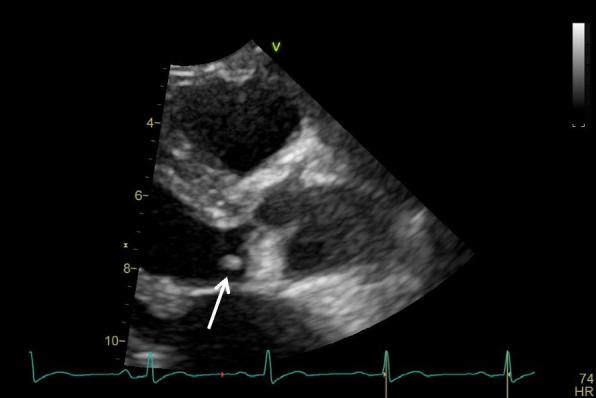
Image of transthoracic echocardiography (TTE). On the TTE, a vegetation (9.0 × 3.0 mm) on the noncoronary cusp of the aortic valve (white arrow) was identified

During his hospitalization, repeated blood cultures taken on the second day showed no bacterial growth after several days, yet the patient continued to be persistently febrile. Based on these findings, a transthoracic echocardiography was performed, revealing a vegetation (9.0 × 3.0 mm) on the noncoronary cusp of the aortic valve and moderate aortic regurgitation, which led to infectious disease consultation (Fig. 2). The visualized valvular vegetation combined with disseminated lesions in his lower back and RF positivity confirmed a definitive diagnosis of IE using the modified Duke criteria [9]. Empiric ampicillin/sulbactam 3.0 g every six hours and vancomycin were started on hospital day ten.

Despite conducting seven sets of blood cultures (including three sets before antibiotic initiation), no bacterial growth was detected. Br-PCR testing for 16S ribosomal RNA (rRNA) using 27 F and 1492R primers, which are commonly used for bacterial identification due to their broad-range specificity, followed by nanopore sequencing, was conducted on the blood and hepatocytes obtained via random liver biopsy due to his acute liver injury. Unfortunately, this failed to identify the causative organism [10, 11]. Additionally, needle aspiration of the iliopsoas muscle and joint was unsuccessful due to insufficient fluid volume.

**Fig. 3:**
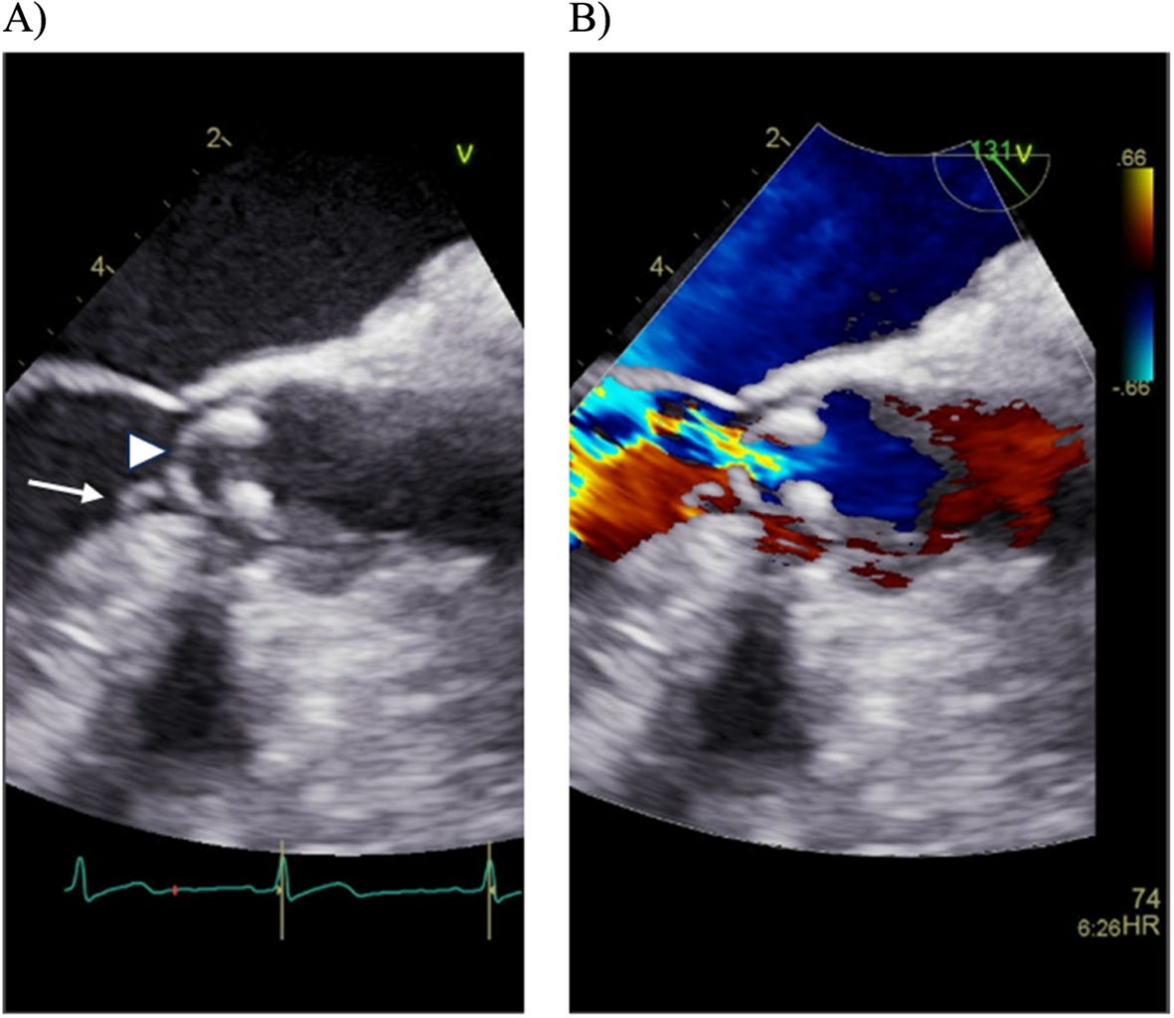
Images of transesophageal echocardiography (TEE). On the TEE, a vegetation (4.8 × 2.8 mm) on the noncoronary cusp (white arrow) and aneurysmal enlargement of noncoronary cusp with perforation (white arrow head) were identified (**A**). A severe aortic regurgitation from perforated noncoronary cusp was demonstrated by TEE with Color Doppler echocardiography (**B**)

**Fig. 4:**
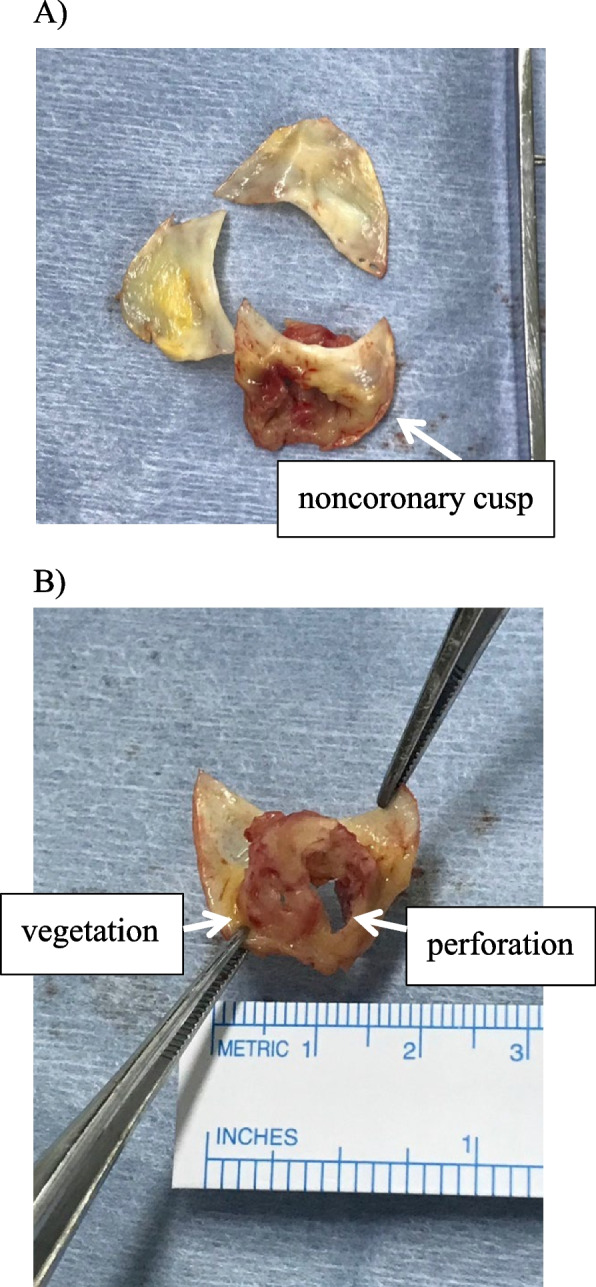
Surgically removed aortic valve. **A** The aortic valve after surgical resection. **B** The visible vegetation and perforation at the noncoronary cusp

**Fig. 5:**
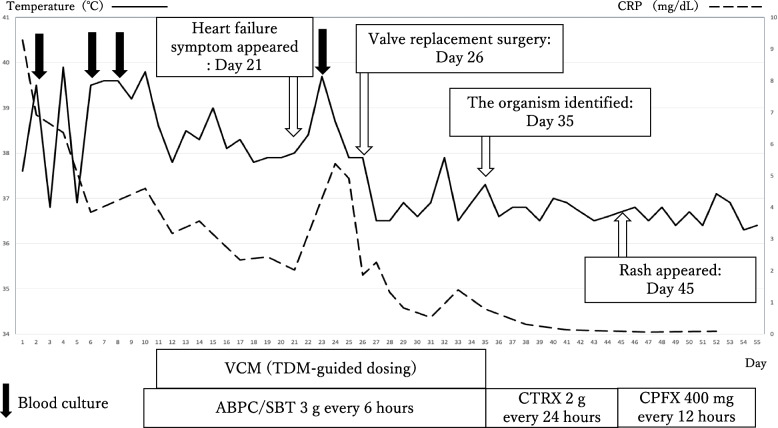
Hospital course of this case. It revealed that fever resolution was obtained promptly after the surgery. CRP: C-reactive protein, ABPC/SBT: ampicillin/sulbactam; VCM: vancomycin; TDM: Therapeutic Drug Monitoring; CTRX: ceftriaxone; CPFX: ciprofloxacin

On hospital day 21, he developed worsening dyspnea, increased respiratory rate, decreased oxygen saturation, and wheezing. A repeat chest X-ray showed new pleural effusions. Transesophageal echocardiography (as a preoperative examination) confirmed a vegetation (4.8 × 2.8 mm) on the noncoronary cusp causing severe aortic regurgitation, and revealed an aneurysm at the noncoronary cusp of the aortic valve with perforation, which was not visible on prior transthoracic echocardiography (Fig. 3). Aortic valve replacement surgery was performed on hospital day 26 due to heart failure progression caused by the severe aortic regurgitation with aortic valve perforation (Fig. 4). His fever and symptoms quickly resolved post-operatively. Although cultures of the excised aortic valve showed no bacterial growth, *A. aphrophilus* was identified by 16S rRNA br-PCR testing of the valve tissue on the 35 th day of admission. This allowed the antibiotics to be de-escalated to ceftriaxone (2.0 g every 24 h). On day 45, a drug-induced skin rash appeared, and the antibiotic regimen was changed to ciprofloxacin (400 mg every 12 h). The patient received four weeks of post-operative antibiotics and remained afebrile. He was discharged on hospital day 55 after completion of intravenous antibiotics without evidence of recurrent infection or surgical complications. Although follow-up imaging was not performed, the patient’s lower back pain gradually improved with antibiotic therapy and fully resolved after aortic valve replacement. At discharge, no musculoskeletal symptoms remained, and no recurrence of infection was observed during follow-up. The hospital course is summarized in Fig. 5.

## Discussion

We present the first known case of culture-negative IE by *A. aphrophilus* presenting with facet joint arthritis as the primary manifestation in a patient without documented cardiac disease. This case is noteworthy for several reasons and has important implications for the diagnosis and management of IE.

### Unusual clinical presentation

Although *A. aphrophilus* IE is generally associated with patients who have preexisting valvular disorders or prosthetic valves [7], our patient had a healthy native valve. To the best of our knowledge, only six prior cases of native valve endocarditis caused by *A. aphrophilus* in patients without cardiac risk factors have been reported, which are summarized in Table 1 [3–6, 12, 13]. In those cases, the predominant clinical manifestations were fever and chills. In contrast, our patient presented with prominent back pain due to facet joint arthritis—a presentation not previously documented. This unusual symptomatology underscores the need for clinicians to consider IE even when patients present primarily with musculoskeletal complaints, particularly when accompanied by persistent fever.

**Table 1:** Comparative table of previous studies of native valve endocarditis caused by *A. aphrophilus*

References’number	First author	Patient	Possible entries	Chief complain	Extra-cardiaccomplications	Blood culture	Cardiac images findings	Antimicrobial therapy	Valvular tissue culture	Valvular tissue PCR	Outcome
	Our Case	58-year-old Male	poor dental condition	fever, fatigue, and persistent lower back pain	facet joint arthritis	all negative	vegetations on the aortic valve and significant regurgitation (TTE)	ABPC/SBT 3 g every 6 h and VCM → CTRX 2 g every 24 h → CPFX 400 mg every 12 h total 6 weeks	surgical case → not detected	detected	Resolved
[3]	Liao, Yu	47-year-old Male	-	fever, chills, and decreased urine output	Roth's spot	all negative	vegetations on the mitral valve (TEE)	CTRX and VCM → CPFX and TEIC → DAP and ERT Duration: not mentioned	surgical case → not detected	detected	Resolved
[4]	Hirano, Koji	72-year-old Female	poor dental condition	fever and hematouria	ANCA-associated vasculitis and mycotic cerebral embolism	all negative	vegetations on the mitral valve and significant regurgitation (TTE)	CTRX and VCM for 4 weeks → CTRX for 4 more weeks	surgical case → not detected	detected	Resolved
[5]	Bagheri, Sina	74-year-old Male	skin or oral cavity	right shoulder pain, generalized weakness, chills, palpitations, and poor appetite	right shoulder abscess, complicated by septic embolization, leading to intracranial hemorrhage	positive	vegetation on the mitral valve and moderate mitral regurgitation (TEE)	ABPC/SBT 3 g every 6 h → GM 1 mg/kg every 8 h added → CTRX 2 g every 24 h for 3 more weeks	-	-	Stabilized
[6]	Daoud, Hussein	53-year-old Male	-	confusion and word finding difficulty, fevers, night sweats, chills, and unintentional weight loss	brain abscess	positive	a flail posterior leaflet with severe regurgitation but no clear vegetation (TEE)	CTRX, VCM and ACV → CTRX for 6 weeks	-	-	Resolved
[12]	Jung, Gordon W	42-year-old Female	poor dental condition	reduced level of consciousness, and incontinence of urine	multiple cerebral abscesses and pulmonary abscess in the left lower lobe	all negative but pleural fluid culture positive	mobile vegetative mass on the noncoronary cusp of the aortic valve (TEE)	CTRX 2 g every 12 h, VCM 1 g every 12 h, and MNZ 500 mg every 8 h	-	-	dead
[13]	Wright, P	51-year-old Male	benign tongue lesion	general malaise with vomiting, diarrhoea, fever, sweats and myalgia	embolic stroke and digital infarction	positive	moderate aortic regurgitation but no obvious vegetations (TEE)	CTRX total 6 weeks	-	-	Resolved

We conducted a search on PubMed to determine the number of cases of endocarditis caused by *A. aphrophilus* previously reported in the past. We found six cases of native valve endocarditis without known cardiac disease reported in the past 20 years

*PCR* polymerase chain reaction, *TTE* transthoracic echocardiography, *TEE* transesophageal echocardiography, *ABPC/SBT* ampicillin/sulbactam, *VCM*: vancomycin, *CTRX* ceftriaxone, *CPFX* ciprofloxacin, *TEIC* teicoplanin, *DAP* daptomycin, *ERT* ertapenem, *MNZ* metronidazole, *GM* gentamicin, *ACV* acyclovir

### Diagnostic challenges and the role of br-PCR

A major challenge in our case was the failure of conventional diagnostic methods. Despite multiple blood cultures and br-PCR testing on both blood and liver tissue, the causative organism remained undetected. Only the br-PCR performed on the excised aortic valve tissue provided a definitive diagnosis by identifying *A. aphrophilus*. This observation reinforces previous reports that suggest br-PCR on heart valve tissue is often essential for pathogen identification in culture-negative IE [2–4]. Our decision to include liver tissue in the diagnostic workup, although ultimately yielding a negative result, represents an attempt to use a less invasive method when direct access to the valve is not possible. However, the negative liver result highlights that elevated transaminase levels do not necessarily correlate with a successful molecular diagnosis. Therefore, this case demonstrates the current limitations of non-invasive diagnostic modalities and emphasizes the critical role of valve tissue br-PCR in such scenarios.

### Therapeutic implications

The clinical course of this patient further illustrates the limitations of empirical antibiotic therapy in culture-negative IE. In our patient, broad-spectrum antibiotics did not lead to clinical improvement until surgical intervention achieved effective source control. The subsequent de-escalation of antibiotics based on the molecular identification of *A. aphrophilus* not only optimized therapy but also minimized unnecessary broad-spectrum antimicrobial exposure. Figure 5 clearly illustrates the initial lack of response to empirical therapy followed by rapid clinical improvement after valve replacement. This case therefore supports the notion that early surgical intervention, combined with targeted antibiotic therapy based on molecular diagnostics, may be necessary in severe cases of culture-negative IE.

### Association with dental etiology and limitations

It is well recognized that *A. aphrophilus* is frequently linked to poor dental hygiene or recent dental procedures, as the organism typically originates from the oral cavity and tongue lesions [4, 5, 12, 13]. Interestingly, our patient did not exhibit significant dental disease, broadening the clinical spectrum of this pathogen.

There are several limitations to our report. First, although *A. aphrophilus* endocarditis is often associated with a favorable prognosis [5, 6, 12], the severity of our patient’s condition necessitated surgical intervention. Second, while br-PCR is invaluable for identifying the causative organism, it does not provide antimicrobial susceptibility data, which is crucial for optimizing targeted therapy. Third, the technical difficulty encountered during the fluoroscopic-guided aspiration of the facet joint—due to a small fluid pocket—prevented preoperative bacterial identification from that site, thereby underscoring the challenges associated with diagnosing IE in the context of atypical presentations.

### Patient outcome and future considerations

Despite these challenges, the patient’s clinical improvement following valve replacement and targeted antibiotic therapy was remarkable. His postoperative course not only alleviated his initial anxiety about persistent back pain and fever but also underscored the importance of pathogen identification in guiding effective treatment. Furthermore, upon learning that the causative organism likely originated from the oral cavity, the patient recognized the importance of maintaining good dental hygiene and expressed a commitment to improving his lifestyle habits.

In summary, our case highlights an unusual presentation of culture-negative *A. aphrophilus* IE with facet joint arthritis in a patient without preexisting cardiac disease. It emphasizes the diagnostic value of valve tissue br-PCR when conventional methods fail and underscores the need for a comprehensive diagnostic and therapeutic approach that includes early surgical intervention. These findings have significant implications for clinical practice, suggesting that a high index of suspicion for IE should be maintained even in patients presenting with unexplained musculoskeletal pain.

## Conclusions

We report the first known case of facet joint arthritis as the initial manifestation of culture-negative native valve IE caused by *A. aphrophilus* in a patient without known cardiac disease. Our case highlights the importance of considering IE in patients with fever and unexplained musculoskeletal pain, even without known cardiac disease. When conventional diagnostic tests fail, br-PCR on excised heart valve tissue remains a critical tool for confirming culture-negative IE. In severe cases requiring surgical intervention, early valve resection should be considered for effective source control. Adherence to current ESC guidelines is essential for early diagnosis and effective treatment [14].

## Data Availability

No datasets were generated or analysed during the current study.

## Abbreviations

*A. aphrophilus*: *Aggregatibacter aphrophilus*
IE: Infective endocarditis
br-PCR: Broad-range polymerase chain reaction
RF: Rheumatoid factor
MRI: Magnetic resonance imaging
STIR: Short tau inversion recovery
rRNA: Ribosomal RNA
